# Eicosopentaneoic Acid and Other Free Fatty Acid Receptor Agonists Inhibit Lysophosphatidic Acid- and Epidermal Growth Factor-Induced Proliferation of Human Breast Cancer Cells

**DOI:** 10.3390/jcm5020016

**Published:** 2016-01-26

**Authors:** Mandi M. Hopkins, Zhihong Zhang, Ze Liu, Kathryn E. Meier

**Affiliations:** Department of Pharmaceutical Sciences, College of Pharmacy, Washington State University, Spokane, WA 99163, USA; mandi.hopkins@wsu.edu (M.M.H.); juliazhang2013@gmail.com (Z.Z.); zeliu1010@gmail.com (Z.L.)

**Keywords:** breast cancer, lysophosphatidic acid, epidermal growth factor, ω-3 fatty acids, G protein-coupled receptors, free fatty acid receptors

## Abstract

Many key actions of ω-3 (*n*-3) fatty acids have recently been shown to be mediated by two G protein-coupled receptors (GPCRs) in the free fatty acid receptor (FFAR) family, FFA1 (GPR40) and FFA4 (GPR120). *n*-3 Fatty acids inhibit proliferation of human breast cancer cells in culture and in animals. In the current study, the roles of FFA1 and FFA4 were investigated. In addition, the role of cross-talk between GPCRs activated by lysophosphatidic acid (LPA), and the tyrosine kinase receptor activated by epidermal growth factor (EGF), was examined. In MCF-7 and MDA-MB-231 human breast cancer cell lines, both LPA and EGF stimulated proliferation, Erk activation, Akt activation, and CCN1 induction. LPA antagonists blocked effects of LPA and EGF on proliferation in MCF-7 and MDA-MB-231, and on cell migration in MCF-7. The *n*-3 fatty acid eicosopentaneoic acid inhibited LPA- and EGF-induced proliferation in both cell lines. Two synthetic FFAR agonists, GW9508 and TUG-891, likewise inhibited LPA- and EGF-induced proliferation. The data suggest a major role for FFA1, which was expressed by both cell lines. The results indicate that *n*-3 fatty acids inhibit breast cancer cell proliferation via FFARs, and suggest a mechanism involving negative cross-talk between FFARS, LPA receptors, and EGF receptor.

## 1. Introduction

Our group recently demonstrated that the inhibitory effects of ω-3 fatty acids on prostate cancer cell proliferation are mediated by FFA4, a G protein-coupled receptor in the free fatty acid receptor (FFAR) family [1]. The purpose of the current study was to determine whether FFARs mediate similar inhibitory effects in human breast cancer cells.

The dietary polyunsaturated ω-3 fatty acids (*n*-3 FAs) are alpha-linolenic acid (ALA), docosahexaenoic acid (DHA) and eicosapentaneoic acid (EPA). Although effects of *n*-3 FAs in prostate cancer have been debated [2], there is relatively strong evidence supporting a preventative effect of *n*-3 FA consumption on many human cancers [3], including breast cancer [4]. Multiple reports show that *n*-3 fatty acids inhibit growth of breast cancer cells, either in cell culture [5,6], or in xenograft tumors [7,8,9,10].

The prevailing mechanistic paradigm has been that *n*-3 FAs exert anti-inflammatory and potentially anti-cancer effects by competitively reducing production of eicosanoids, and/or more directly by generating metabolites with anti-inflammatory activity (e.g., “resolvins”) [11,12]. However, the direct effects of *n*-3 metabolites on cancer cells, as compared to their anti-inflammatory effects, are under-studied [13]. In one report, resolvin D2, a DHA metabolite, unexpectedly increased proliferation of MCF-7 breast cancer cells [14].

Alternatively, it was shown in the last decade that *n*-3 FAs are agonist ligands for free fatty acid receptors (FFARs) that were formerly “orphan receptors” [15]. Two G-protein-coupled receptors (GPCRs), FFA1 and FFA4, bind long-chain polyunsaturated fatty acids that include *n*-3 FAs. The “de-orphanization” discovery has led to the ongoing characterization of the roles of the FFARs in cellular regulation, and to the rapid development of selective FFAR agonists with therapeutic potential [16,17,18,19].

Several studies have specifically explored the mechanism of the inhibitory action of *n*-3 FAs on breast cancer cells. The pathways implicated in the response include decreased Akt activation [5], increased neutral sphingomyelinase activity [20], increased BRCA levels [21], and increased PTEN levels [22]. GPCR-independent mechanisms have been reviewed [23]. To date, there is little information available concerning the roles of FFARs in breast cancer. It has however been shown that FFA1 is expressed in MCF-7 cells [24], and that MCF-7 and MDA-MB-231 cells express both FFA1 and FFA4 [25,26].

One study investigated the role of FFA4 in breast cancer in a mouse model, focusing on the role of FFA4 in inhibiting inflammation [27]. In this study, *n*-3 FAs reduced tumor burden even when FFA4 was knocked out in the host mouse. The authors suggest that anti-inflammatory effects of *n*-3 FAs, mediated by FFA4, are not important for their anti-tumor effects. Using cultured cells derived from their mouse model, the investigators showed that DHA induced apoptosis in either wild-type or FFA4 knockdown cells when applied at high doses (40–100 µM). The role of the alternative *n*-3 FA receptor, FFA1, has not been examined in breast cancer cell, to our knowledge.

In this study, we utilized two commonly used human breast cancer cell lines: MCF-7 and MDA-MB-231, as experimental models. MCF-7 is a luminal A estrogen receptor positive cell line, while MDA-MB-231 is a highly metastatic triple negative cell line. These two cell lines were used to explore the role of FFARs in the mechanism of action of *n*-3 FAs in breast cancer.

## 2. Experimental Section

### 2.1. Materials

EPA (prepared in ethanol) was from Cayman Chemical (Ann Arbor, MI, USA). The FFAR agonists TUG-891 (4-[(4-fluoro-4′-methyl[1,1′-biphenyl]-2-yl)methoxy]-benzenepropanoic acid; prepared in dimethylsulfoxide (DMSO) and GW9508 (4-[[(3-phenoxyphenyl)methyl]amino]-benzenepropanoic acid; prepared in ethanol), were from Millipore (Billerica, MA, USA) and Cayman Chemical, respectively. AM966 (2-[4-[4-[4-[[(1*R*)-1-(2-chlorophenyl)ethoxy]carbonylamino]-3-methyl-1,2-oxazol-5-yl]phenyl] phenyl]acetic acid) was purchased pre-dissolved in DMSO from MedChem Express (Monmouth Junction, NJ, USA). Ki16425 (3-[[[4-[4-[[[1-(2-chlorophenyl)ethoxy]carbonyl]amino]-3-methyl-5-isoazoly]phenyl]methyl]thio]-propanoic acid; prepared in DMSO) was purchased from Cayman Chemical (Ann Arbor, MI, USA). Vehicle controls were included in all samples not receiving FFAR agonists or LPA receptor (LPAR) antagonists (final concentrations of 0.03% (*v*/*v*) ethanol or 0.01% DMSO). LPA (18:1; oleoyl) was obtained from Avanti Polar Lipids (Birmingham, UK), and was delivered to cells as a 1000X stock solution prepared in 4 mg/mL fatty acid-free bovine serum albumin (BSA). The vehicle control for LPA was a final concentration of 4 µg/mL BSA. EGF was from Sigma (St. Louis, MO, USA). Antibody recognizing CCN1 (lot # F0509; 1:1000 dilution) was obtained from Santa Cruz Biotechnology (Santa Cruz, CA, USA). Anti-actin, obtained from BD Transduction Laboratories (Lexington, KY, USA) (lot # 51711), was used at a 1:5000 dilution. Goat anti-rabbit secondary antibody (lot #083M4752) was purchased from Sigma (St Louis, MO, USA) and used at 1:20,000 dilution, while goat anti-mouse secondary antibody (lot #1124907A) was purchased from Invitrogen/Life Technologies (Grand Island, NE, USA) and used at a 1:5000 dilution.

### 2.2. Cell Culture

MCF-7 and MDA-MB-231 cells were obtained from the American Type Culture Collection (Manassas, VA, USA). The cells were grown in RPMI medium supplemented with 10% FBS (Hyclone/Thermo-Fisher Scientific, Waltham, MA, USA). Both cell lines were grown in an incubator at 37 °C in 5% CO_2_ on standard tissue culture plastic.

### 2.3. Cell Proliferation Assays

*C*ells were seeded in 6-well plates at 3 × 10^5^ cells/well in serum-containing medium. After 1 day, the medium was changed to RPMI 1640 without serum. On the next day, the medium was changed to RPMI 1640 with 10% FBS, 10 µM LPA, or 10 nM EGF, in the absence or presence of 100 nM AM966, 10 µM Ki16425, 20 µM EPA or 1 µM TUG-891. Control cells were incubated with the appropriate vehicle (0.03% ethanol, *v*/*v*; 0.01% DMSO, *v*/*v*; and/or 4 µg/mL BSA). Duplicate wells were prepared for each experimental condition. Cell numbers were evaluated after 24, 48, and 72 h by removing medium, incubating cells with trypsin/EDTA for 5 min, adding trypan blue, and counting the suspended live cells (excluding trypan blue) using a hemacytometer.

### 2.4. Cell Migration Assays

MCF-7 cell migration was assessed using a modified Boyden chamber method, as previously described [28]. Cells were serum starved for 24 h and then seeded in serum-free medium at 2.5 × 10^4^ cells per insert in the upper chambers of 8-µm transwell inserts (BD Biosciences, San Jose, CA, USA). Cells were then treated with 10% FBS, 100 nM AM966, 20 µM EPA, 1 µM TUG-891, 10 µM LPA, or 10 nM EGF, either alone or in combination, with appropriate vehicle controls as described above. Serum-free medium was added to the lower wells. Following a 6-h migration, the insert membranes were fixed and stained using methanol and crystal violet. Cells that invaded the lower chambers were counted by microscopy.

### 2.5. Cell Incubations for Signal Transduction Assays

Cells were grown in DME medium supplemented with 10% FBS until ~80% confluent. Cells were serum-starved for 24 h in RPMI 1640 medium, then incubated with 10 µM LPA, 10 nM EGF, and/or 100 nM AM966 or 10 µM Ki16425 for 10 min. Cells were rinsed twice with ice-cold phosphate-buffered saline (PBS), harvested by scraping into 1 mL ice-cold PBS, collected by centrifugation at 10,000× *g* for 10 min at 4 °C, and resuspended in ice-cold lysis buffer (20 mM HEPES (pH = 7.4)), 1% Triton X-100, 50 mM NaCl, 2 mM EGTA, 5 mM β-glycerophosphate, 30 mM sodium pyrophosphate, 100 mM sodium orthovanadate, 1 mM phenylmethylsulfonyl fluoride, 10 µg/mL aprotinin, 10 µg/mL leupeptin). Insoluble debris was removed after centrifugation.

### 2.6. Reverse Transcription Polymerase Chain Reacton (RT-PCR)

For analysis of FFAR expression, total RNA was isolated using an RNeasy Mini kit (Qiagen, Valencia, Spain). First-strand complementary DNA (cDNA) was synthesized with SuperScript II Reverse Transcriptase (Invitrogen) following the manufacturer’s instructions using 20 µL of reaction mixture containing 2 µg RNA. PCR was carried out using Platinum *Pfx* DNA Polymerase (Invitrogen) and Integrated DNA Technology (San Diego, CA, USA) primers: FFA4 (F: 5′-CCTGGAGAGATCTCGTGGGA-3′; and R: 5′-AGGAGGTGTTCCGAGGTCTG-3′); FFA1 (F: 5′-CTCCTTCGGCCTCTATGTGG-3′; and R: 5′-AGACCAGGCTAGGGGTGAGA-3′); RPLP0 (F: 5′-CGCTATCCGCGGTTTCTGAT-3′; and R: 5′-AGACGATGTCACTTCCACGA-3′). For each reaction, 5 µg cDNA template was used. Products were separated by ethidium bromide agarose gel electrophoresis, and were then imaged using a ChemiDoc with Image Lab software (Bio-Rad, Hercules, CA, USA). For analysis of LPA receptor expression, total RNA was extracted from harvested cells using TRIzol solution (Invitrogen, Carlsbad, CA, USA) according to the manufacturer’s protocol. Reverse transcription was performed using iScriptTMcDNA synthesis kit (Bio-Rad, Hercules, CA) in a reaction volume of 20 µL under the conditions recommended by the manufacture. Total RNA (1 µg) was used as a template for cDNA synthesis. PCR was performed in a 50-µL reaction volume with a buffer consisting of 10 × iTaq buffer, 50 mM MgCl_2_, 10 mM dNTP mix, iTaq DNA polymerase;and 0.25 µmol/L each primer. The primers were: LPA1/Edg-2 (F: 5′-TGTCATGGCTGCCATCTC-3′; and R: 5′-CATCTCAGTTTCCGTTCTAA-3′); LPA2/Edg-4 (F: 5′-CCCAACCAACAGGACTGACT-3′; and R: 5′-GAGCCCTTATCTCTCCCCAC-3′); LPA3/Edg-7 (F: 5′-GGACACCCATGAAGCTAATG-3′; and R: 5′-TCTGGGTTCTCCTGAGAGAA-3′); β-actin (F: 5′-TGACGGGGTCACCCACACTGTGCCCATCTA-3′; and R: 5′-CTAGAAGCATTTGCGGTGGACGATGGAGGG-3′). RT-PCR products were separated on a 2% agarose gel by electrophoresis and visualized and imaged under UV illumination.

### 2.7. Immunoblotting

Whole-cell extracts containing equal amounts of protein (30 µg) were separated by SDS-PAGE on 10% Laemmli gels, transferred to nitrocellulose, and incubated with primary (overnight at 4 °C) and then secondary (one to two hours at room temperature) antibodies. Blots were developed using enhanced chemiluminescence (GE Healthcare, Pittsburgh, PA, USA), and imaged using a Gel Doc system (BioRad, Hercules, CA, USA). Protein expression was quantified by densitometry using Quantity One software (Bio-Rad). Results were normalized to the actin loading control, and then to the value obtained for untreated control cells.

### 2.8. Statistical Analysis

Data were analyzed by two-way ANOVA followed by Tukey’s multiple comparisons test. The only exceptions were assays in which there was only one time point (e.g., migration assays); these data were analyzed by one-way ANOVA followed by Tukey’s mutliple comparisons test. All analyses were done using Prism software (Graphpad, San Diego, CA, USA).

## 3. Results and Discussion

### 3.1. Effects of Lysophosphatidic Acid (LPA) and Epidermal Growth Factor (EGF) on Breast Cancer Cell Proliferation

**Figure 1:**
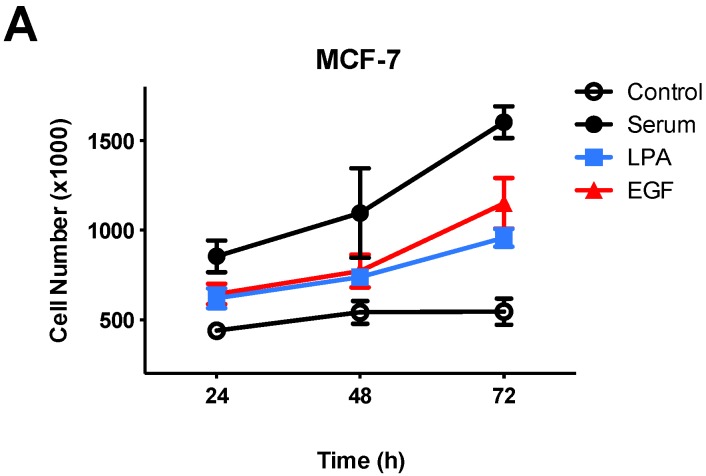

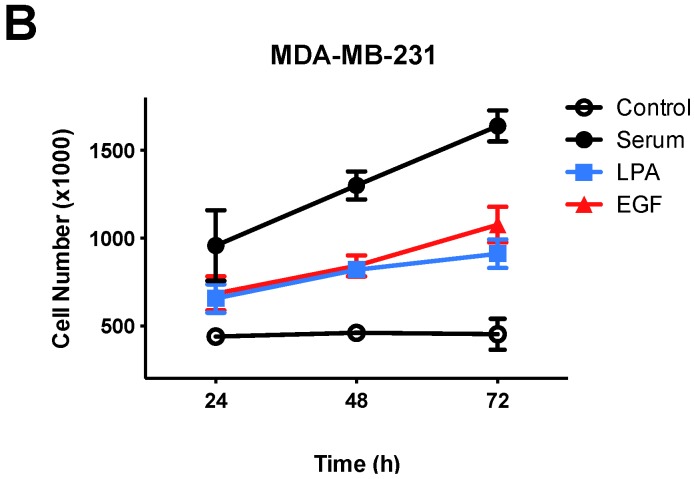
Effects of growth factors on proliferation of human breast cancer cells. Proliferation assays were conducted using serum-starved MCF-7 (**A**) or MDA-MB-231 (**B**) cells. Cells were incubated with or without 10% FBS (serum), 10 µM LPA, or 10 nM EGF for the indicated times (growth factors were added at time “0”). Each data point represents the mean ± SEM (*n* = 4) of values (number of live cells per well) from two separate experiments, each done in with two separate replicate wells of cells for each condition. Data analysis was performed using two-way ANOVA, followed by Tukey’s multiple comparisons test. All growth factor values were significantly (*p* < 0.05) different from the control value at all time points shown, except for LPA at 24 h in MCF-7. Serum values were significantly different from lysophosphatidic acid (LPA) or epidermal growth factor (EGF) at all time points tested.

Before testing for effects of FFAR agonists on breast cancer cells, we first established conditions for using growth factors to stimulate proliferation. Cells were serum-starved before treatments in order to remove confounding effects of LPA contained in serum, and to provide a baseline for testing effects of growth factors. The effects of serum, LPA, and EGF on proliferation of serum-starved MCF-7 and MDA-MB-231 cells are shown in Figure 1. All growth factors significantly increased cell number as compared to control. Serum was significantly more effective in inducing proliferation than LPA or EGF at all time points tested, in both cell lines; this result was expected since serum contains multiple mitogens including LPA. There was no significant difference between responses to LPA *versus* EGF at any time point.

### 3.2. Signal Transduction Responses to LPA and EGF in Breast Cancer Cells

**Figure 2:**
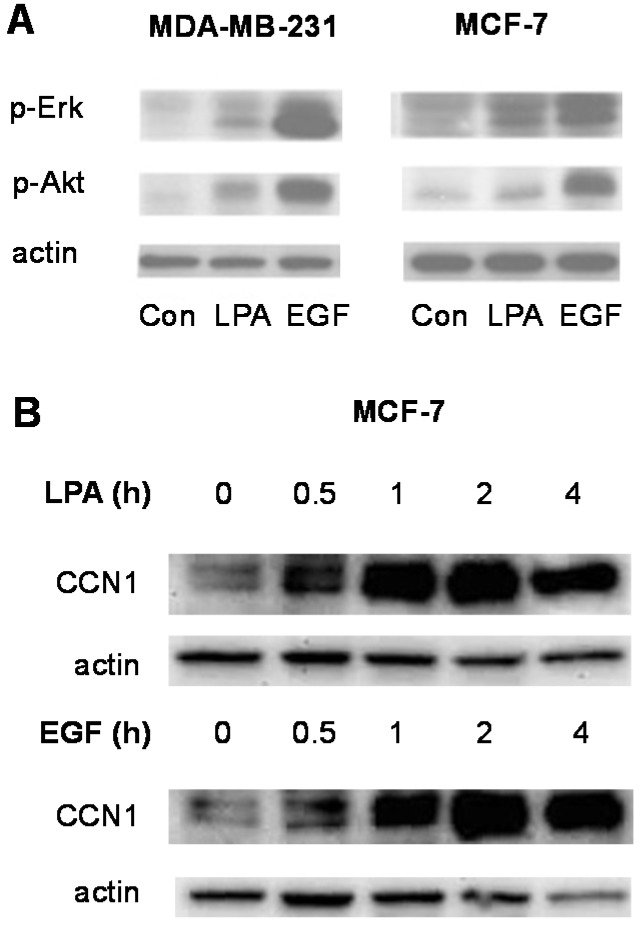
Effects of LPA and EGF on signal transduction events in human breast cancer cells. (**A**) Serum-starved MCF-7 and MDA-MB-231 cells were incubated with 10 µM LPA or 10 nM EGF for 5 min. Whole-cell extracts were immunoblotted using antibodies recognizing phosphorylated active Erk and Akt. An immunoblot for total actin was performed as a loading control; (**B**) Serum-starved MCF-7 cells were incubated for the indicated times with 10 µM LPA or 10 nM EGF. Whole-cell extracts were immunoblotted using antibody recognizing total CCN1. An immunoblot for total actin was performed as a loading control. Each experiment is representative of at least three separate experiments.

To further characterize responses to LPA and EGF in the breast cancer cell lines, we performed immunoblotting experiments to test for Erk and Akt activation (Figure 2). Both LPA and EGF increase activating phosphorylation of Erk and Akt in both MCF-7 and MDA-MB-231 cells (Figure 2A). These results are consistent with previous reports concerning the mitogenic activity of LPA and EGF in breast cancer cells [29,30].

We also examined a more novel response to growth factors, CCN1 induction, in MCF-7 cells. CCN1 is an inducible matricellular protein whose expression is positively correlated with breast cancer progression [31,32]. As shown in Figure 2B, both LPA and EGF stimulate expression of CCN1 in MCF-7 cells. An increase in CCN1 protein levels was seen after only 30 min of treatment with either growth factor. Taken together, the results presented in Figure 2A,B confirm that both LPA and EGF activate pro-mitogenic signaling pathways in human breast cancer cell lines.

### 3.3. Effects of LPA Antagonists on Breast Cancer Cell Proliferation

**Figure 3:**
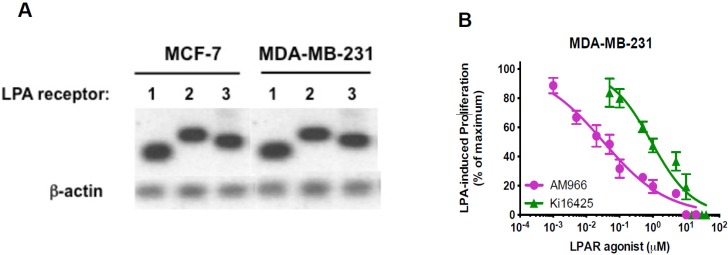
LPAR expression in breast cancer cell lines, and dose-response for the effects of LPA_1_ antagonists on MDA-MB-231 cell proliferation. (**A**) Total RNA was extracted from MCF-7 and MDA-MB-231 breast cancer cells. RT-PCR was performed (separate gels for each cell line) using the cDNA primers described in the Methods. β-actin was amplified as loading control; (**B**) Serum-starved MDA-MB-231 cells were incubated for 48 h with and without 10 µM LPA in the absence and presence of the indicated concentrations of Ki16425 or AM966. The number of cells achieved in response to LPA alone was defined as 100% response; the number of cells in the absence of LPA was defined as 0% response. Each point represents mean ± SEM (*n* ≥ 4) for values obtained from at least two experiments, each performed with two separate replicate wells of cells for each condition.

Previous studies have shown that LPA receptors are expressed in breast cancer cell lines. One group performed a comprehensive analysis of LPA receptor expression in commonly-used breast cancer cell lines [33]. Their data show that MDA-MB-231 and MCF-7 cells, among other cell lines, express LPA_1_, LPA_2_, and LPA_3_. LPA_1_ has been determined to mediate many of the actions of LPA in breast cancer cells [34,35,36,37]. RT-PCR experiments conducted in our lab confirmed that LPA_1_ was expressed in both MCF-7 and MDA-MB-231 cells under the conditions used herein (Figure 3A).

Two LPA receptor pharmacologic antagonists were used to further study the role of LPA_1_ in breast cancer cells. Ki16425 is a selective inhibitor of LPA_1_ and LPA_3_ [38], while AM966 is an LPA_1_-selective antagonist [39]. We used a dose-response study (Figure 3B) to test whether LPA_1_ inhibitors inhibit LPA-induced proliferation in MDA-MB-231 breast cancer cells. As expected, based on their relative receptor affinities, the LPA_1_-selective antagonist AM966 was more potent (IC_50_ = 32 nM) in inhibiting LPA-induced proliferation than was the pan-LPA inhibitor Ki16425 (IC_50_ = 904 nM). Interestingly, the amount of AM966 needed to completely inhibit MDA-MB-231 cell proliferation was 100-fold higher than in our previously published work using DU145 prostate cancer cells [1], although the amount of Ki16425 required was similar in both MDA-MB-231 and DU145. The dose-response curve for AM966 was very shallow, suggesting the involvement of multiple receptors. These results suggest that LPA_1_ is not the only LPA receptor that mediates LPA-induced proliferation in these cells.

**Figure 4:**
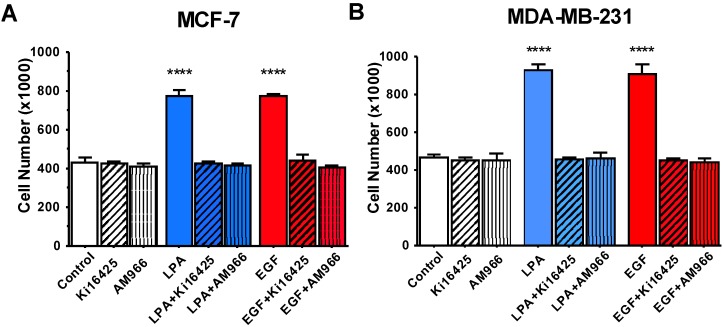
Effects of LPAR antagonists on breast cancer cell proliferation. Serum-starved MCF-7 (**A**) or MDA-MB-231 (**B**) cells were incubated with or without 10 µM LPA or 10 nM EGF in the absence and presence of 10 µM AM966 or 10 µM Ki16425. Each data point represents the mean ± SEM (*n* = 4) of values (number of live cells per well) from two separate experiments, each performed with two separate replicate wells of cells for each experimental condition (**** *p* < 0.0001 compared to control). The 48-h time is point shown; similar results were obtained at 24 and 72 h. Data analysis was performed using two-way ANOVA, followed by Tukey’s multiple comparisons test.

In Figure 4, we further tested the effects of LPA antagonists. We asked 1) whether the inhibitory effects of LPA antagonists extend to EGF-induced proliferation, and 2) whether LPA antagonists have similar effects in MCF-7 and MDA-MB-231 cells. The results of this series of experiments, which are presented in Figure 4, clearly demonstrate that the LPA receptor antagonists block proliferation in response to both LPA and EGF. This response was observed in both MCF-7 and MDA-MB-231 cells.

Taken together, these results are consistent with a crucial role for LPARs in EGF response, as has been noted in other cell types [1,40].

### 3.4. Expression of Free Fatty Acid Receptors (FFARs) in Breast Cancer Cells

**Figure 5:**
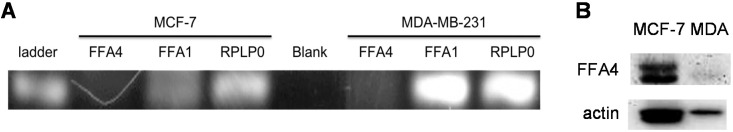
Expression of FFA1 and FFA4 in breast cancer cell lines. (**A**) RT-PCR reactions were carried out for human FFA4, FFA1, or RPLP0 (loading control) as described in Methods. Products were separated and visualized under UV light. Results shown are representative of two separate experiments, each done in triplicate. Faint signals for FFA4 were confirmed in additional experiments; (**B**) Whole-cell extracts, from MCF-7 and MDA-MB-231 cells growing in serum, were separated by SDS-PAGE and then immunoblotted for FFA4 and actin (loading control).

We next turned to the effects of *n*-3 FAs. We examined whether FFARs are expressed in MCF-7 and MDA-MB-231 cells. Both receptors have previously been reported to be present in MCF-7 cells, based on flow cytometry [25]. Using RT-PCR, we assessed mRNA levels for FFA4 and FFA1, the two receptors for long chain free fatty acid (Figure 5A). Both PCR products were detected, although the PCR product for FFA4 was present at such low levels that it was difficult to visualize. To confirm that FFA4 was expressed, we performed immunoblotting using an antibody previously validated in our laboratory [1], and did detect FFA4 protein (Figure 5B). We were unable to validate an appropriate antibody for FFA1, but our PCR results suggest that FFA1 is likely expressed at higher levels than FFA4 in the two breast cancer cell lines. These results indicate that the roles of both FFA1 and FFA4 need to be considered in breast cancer cells.

### 3.5. Effects of FFAR Agonists on Breast Cancer Cell Proliferation

**Figure 6:**
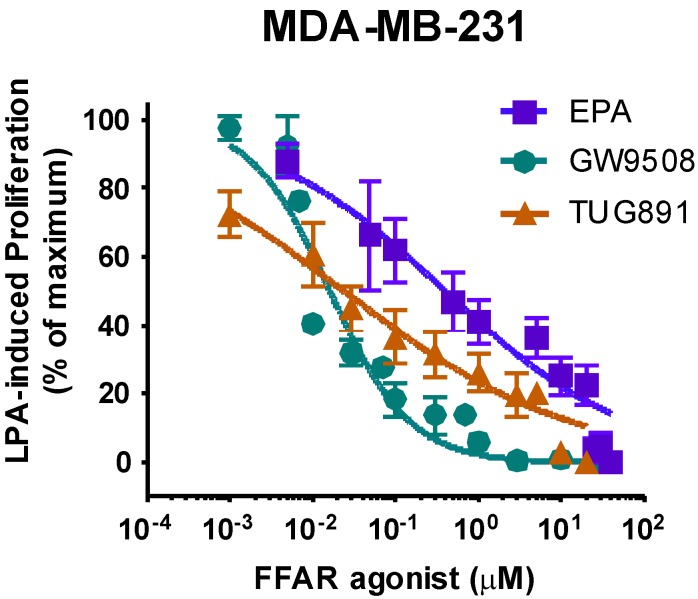
Dose-response for effects of FFAR agonists on breast cancer cell proliferation. Serum-starved MDA-MB-231 cells were incubated for 48 h with and without 10 µM LPA in the absence and presence of the indicated concentrations of EPA, GW9508, and TUG-891. The number of cells achieved in response to LPA alone was defined as 100% response; the number of cells present in the absence of LPA was defined as 0% response. Each point represents mean ± SEM (*n* ≥ 4 for values obtained from at least two experiments, each performed with two separate replicate wells of cells for each experimental condition.

We next performed a dose-response study (Figure 6) to test whether FFAR agonists inhibit proliferation of MDA-MB-231 cells. EPA, TUG-891, and GW9503 all inhibited proliferation of these cells. EPA was the least potent compound (IC_50_ 403 nM), which was expected based on results with other cell lines [1]. The IC_50_ for the FFA4-selective agonist, TUG-891, was 24 nM. However, a 100-fold higher dose of TUG-891 was required to completely inhibit LPA-induced proliferation in the breast cancer cell line (Figure 6) as compared to our previously published results with Du145 prostate cancer cells [1]. In addition, the dose-response curve for TUG-891 was very shallow, suggesting the involvement of more than one receptor in the inhibitory response. While TUG-891 is selective for FFA4 over FFA1, neither TUG-891 nor GW9508 is specific for a single receptor. The FFA1-specific agonist GW9508 was 100-fold more potent (IC_50_ = 16 nm) in inhibiting LPA-induced proliferation in MDA-MB-231 (Figure 6) as compared to previous results in DU145 cells where FFA4 response predominates [1]. The published EC_50_ for GW9508 at FFA1 is ~50 nM. Thus, our results are consistent with a role for FFA1, and possibly also FFA4, in inhibiting proliferation of MDA-MB-231 cells.

**Figure 7:**
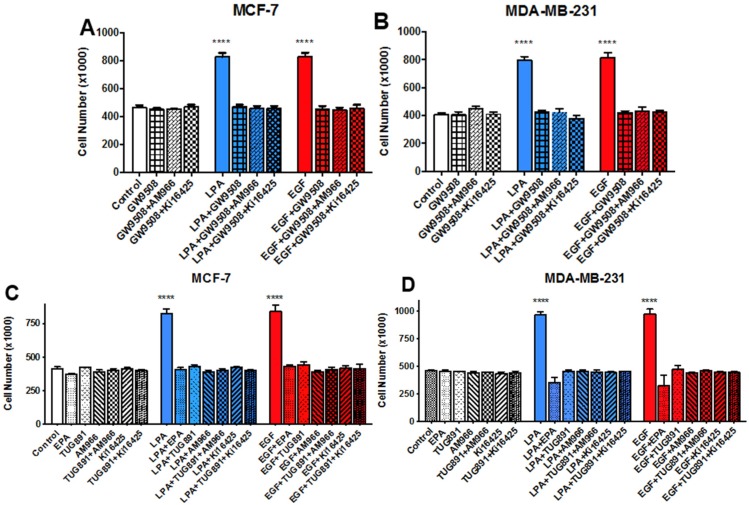
Effects of FFAR agonists on breast cancer cell proliferation. Serum-starved MCF-7 or MDA-MB-231 cells were incubated with or without 10 µM LPA or 10 nM EGF for 48 h in the absence and presence of 1 µM GW9508, 10 µM Ki16425, and/or 10 µM AM966 (Panels **A** and **B**), or (Panels **C** and **D**), 10 µM LPA or 10 nM EGF in the absence and presence of 10 µM TUG891, 10 µM Ki16425, and/or 10 µM AM966. In addition, Panels C and D show the effects of TUG-891 as compared to that of 25 µM EPA, and controls were included for the LPA antagonists alone. Some of the data points from Panels **C** and **D** were presented earlier in Figure 4, which was derived from the same series of experiments; panels (**A**) and (**B**) are from separate sets of experiments. Although only the 48-h time point is shown, similar inhibitory effects were observed at 24 and 72 h. Each value represents the mean ± SEM (*n* = 4) of values (number of live cells per well) obtained from two separate experiments, each of which used two separate replicate wells of cells for each experimental condition (**** *p* < 0.0001 compared to control). Data were analyzed done using two-way ANOVA, followed by Tukey’s multiple comparisons test.

We next tested the effects of LPA_1_ inhibitors on both LPA- and EGF-induced breast cancer cell proliferation (Figure 7). Neither the LPA antagonists AM966 and Ki16425, nor the FFAR agonists EPA, GW9508, and TUG891, had any significant effects on cell numbers on their own. However, all agents completely inhibited LPA- and EGF-induced proliferation in MCF-7 and MDA-MB-231 cells, when added individually, consistent with the results in Figure 4 and Figure 6. Although EPA appeared to decrease cell numbers slightly below control values in the presence of LPA or EGF, the effect was not significant. There was no additional effect (e.g., cytotoxicity) on cell numbers when LPA antagonists were combined with FFAR agonists GW9508 or TUG891 (Figure 7). Thus, both LPA antagonists and FFAR agonists can block the effects of LPA and EGF on breast cancer cell proliferation.

### 3.6. Effects of FFAR Agonists on Breast Cancer Cell Migration

**Figure 8:**
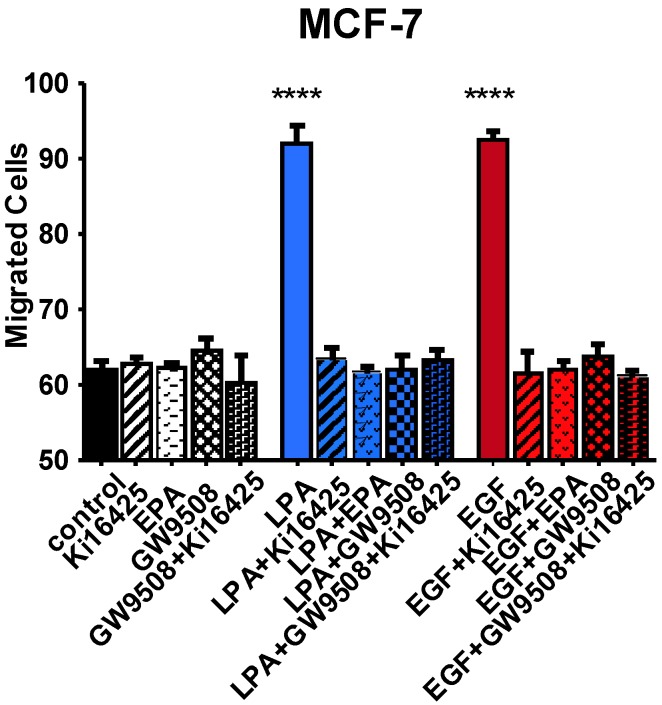
Effects of LPAR agonists on MCF-7 cell migration. Serum-starved MCF-7 cells were treated with 10 µM Ki16425, 20 µM EPA, 1 µM GW9508, 10 µM LPA, or 10 nM EGF, either alone or in combination. After a 6-h migration period, cells were analyzed as described in Methods. Each bar represents the mean ± SEM (*n* = 4) of values (total number of migrated cells/well) obtained from two separate experiments, each of which used two separate replicate wells of cells for each experimental condition (**** *p* < 0.0001 *vs.* control). Data were analyzed using one-way ANOVA, followed by Tukey’s multiple comparisons test.

Finally, we asked whether FFAR agonists inhibit migration of breast cancer cells. As shown in Figure 8, EPA and GW9508 both block LPA- and EGF-induced migration of MCF-7 cells. The LPAR antagonist Ki16425 has a similar effect. The combination of GW9508 and Ki16425 also yields complete inhibition; *i.e.*, there is no additional effect of the FFAR agonist in the presence of an LPA antagonist. We conclude that either activation of FFARs, or antagonism of LPARs, can inhibit migration in response to either LPA or EGF.

## 4. Conclusions

In extending our ongoing studies of FFAR activation to breast cancer cells, the intention was to expand our knowledge of the roles of FFARs in cancer. While others have examined the effects of *n*-3 FAs on breast cancer, to our knowledge no other studies have tested the roles of FFARs in human breast cancer cells.

The current study used LPA as one of the growth factors, based on our previous work implicating LPARs in the mechanism of FFAR-mediated inhibition of cancer cell proliferation. LPA has multiple actions that support breast cancer growth, migration, invasion, and survival [37,41,42,43,44]. An LPA antagonist was shown to induce regression of breast tumors in a mouse model [45]. Others reported that either an LPA_1_ antagonist or LPA_1_ knockdown decreases MDA-MB-231 cell metastasis, but not primary tumor size, in mice [46]. Together, these data have established a potential role for the LPA axis as a therapeutic target in breast cancer cells, as reviewed by Panupinthu and colleagues [29]. However, the overall roles of LPA and its receptors in breast cancer cell proliferation have not been fully delineated.

FFA1/GPR40 was first reported to be present in MCF-7 cells in 2004 [24]. This was later confirmed by another group using flow cytometry [25] and also in the current study by PCR (Figure 5). Soto-Guzman and colleagues also detected FFA4 expression in MCF-7 cells [25]. Our data demonstrate expression of FFA1 and FFA4 mRNA, and FFAR protein, in both MCF-7 and MDA-MB-231 cells. Interestingly, the results of dose-response studies using two selective FFAR agonists were consistent with a major role for FFA1 in MDA-MB-231 cells (Figure 6). While these results do not exclude a role for FFA4, this is the first demonstration in our lab that FFA1 and FFA4 may similarly mediate inhibition of cancer cell proliferation.

Figure 4 and Figure 7 demonstrate that LPAR inhibition not only impedes LPA-induced proliferation, but also EGF-induced proliferation. This result suggests that, in MCF-7 and MDA-MB-231 cells, EGFR is reliant on one or more LPARs. This result is similar to those obtained with prostate cancer cells [1,40]. LPARs have been described as “masters” of EGFR signaling [47], as confirmed by studies in various cell types [1,48,49,50,51,52,53]. Further studies will be required to determine whether FFA1 or FFA4 act directly on both LPARs and EGFR to result in inhibition, or indirectly inhibit EGFR via effects on LPARs.

FFAR activation through EPA, GW9508, or TUG-891 abolishes LPA- and EGF-induced proliferation and migration in MCF-7 and MDA-MB-231 cells (Figure 6, Figure 7 and Figure 8). GW9508 yields a classical dose-response curve with an IC_50_ consistent with action at FFA1. At the higher dose of TUG-891 used to achieve complete inhibition in breast cancer cells (10 µM), it is plausible that it is activating FFA1 in addition to, FFA4. Our results are consistent with other reports of inhibitory effects of *n*-3 FAs in breast cancer cells. In one study, low doses of *n*-3 FAs were shown to inhibit proliferation of MCF-7 cells, while high doses induced apoptosis [20]. This is consistent with our dose-response results for MDA-MB-231 cells, where inhibition of proliferation was achieved without cytotoxicity (Figure 6). The EPA concentration (25 µM) used in subsequent experiments was chosen as the dose that maximally inhibited proliferation but did not decrease viability, since our focus was on inhibition of growth factor response rather than toxic responses that may occur at high doses.

Although our results suggest that both FFAR agonists and LPAR antagonists inhibit proliferation in MCF-7 and MDA-MB-231 cells, more work is needed to fully elucidate the mechanism of action. It remains to be definitively determined which FFAR is responsible for the inhibition in breast cancer cells. It is also unclear at this point whether FFAR activation directly influences EGF-mediated signaling, or whether FFARs act indirectly via interference with LPAR activity as appears to be the case in prostate cancer cells [40]. Since the synthetic FFAR agonists used in the current study are not metabolized to resolvins, and are designed to act selectively at GPCRs, utilization of these agonist drugs is helpful in distinguishing receptor-mediated effects from effects of *n*-3 FAs on lipid metabolism. In addition, the emerging small-molecule FFAR agonists have therapeutic potential for the prevention and/or treatment of breast cancer. In summary, the results presented herein demonstrate for the first time that FFAR activation results in inhibition of breast cancer cell proliferation and migration. The inhibitory response mediated by FFARs needs to be taken into account when considering effects of *n*-3 fatty acids on breast cancer cells.
